# Celiac Disease and Autoimmune Thyroid Disease: The Two Peas in a Pod

**DOI:** 10.7759/cureus.26243

**Published:** 2022-06-23

**Authors:** Tejaswini Ashok, Nassar Patni, Mahejabeen Fatima, Aselah Lamis, Shiza W Siddiqui

**Affiliations:** 1 Internal Medicine, Jagadguru Sri Shivarathreeshwara (J.S.S) Medical College, Mysore, IND; 2 Internal Medicine, Deccan College of Medical Sciences, Hyderabad, IND; 3 Research, Dubai Medical College, Dubai, ARE

**Keywords:** celiac disease–anti-gliadin iga–gluten–gliadin, celiac disease and autoimmunity, gluten-free diet, autoimmune thyroid disorders, celiac disease and thyroid

## Abstract

Celiac disease (CD) is a small intestinal inflammatory disease commonly seen in the Western population. It has been observed that patients with monoglandular and polyglandular autoimmunity have a higher prevalence of celiac disease. Hashimoto's thyroiditis (HT) and Graves' disease (GD), which mainly constitute the autoimmune thyroid diseases (AITD), characterized by lymphocytic infiltration of the thyroid parenchyma, are noted to be frequently associated with celiac disease. The fundamental mechanism of this frequent coexistence is thought to be a shared genetic background. Due to the subclinical nature of the celiac disease, the diagnosis is often missed or made coincidentally during screening. The rising prevalence of the celiac disease among AITD patients has urged researchers to investigate the link between the two. We reviewed the most recent and relevant literature on the intriguing relationship between celiac disease and thyroid autoimmunity. The objectives of this article were to study the shared genetic background, the incidence of CD in AITD, the effect of a gluten-free diet on AITD, and the need for routine screening of CD in AITD patients.

## Introduction and background

Celiac disease (CD) is an immune-mediated enteropathy that occurs in genetically predisposed individuals in response to the consumption of gluten, a protein complex found in wheat and related grains, such as barley, rye, and oats [1]. CD is relatively common in Western populations with a prevalence of around 1%, but the disease is grossly underdiagnosed, partially due to the fact that many cases are asymptomatic but also due to its previously assumed infrequency [1]. It has been reported to occur frequently in patients with certain syndromes and autoimmune disorders, such as Down syndrome, Turner syndrome, type 1 diabetes mellitus, and autoimmune thyroid diseases (AITD) [1]. CD typically presents with gastrointestinal symptoms, apparent malabsorption, weight loss, and/or developmental delay in children. Adults are mostly asymptomatic with positive serum indicators and histology. Atypical features of CD are extraintestinal signs such as herpetiform dermatitis or amelogenesis imperfecta [1]. Many individuals with CD are detected by familial or random screening because of the asymptomatic or silent nature of the disease [1]. Although a small intestine biopsy remains the diagnostic gold standard, highly sensitive and specific serological tests such as tissue transglutaminase (tTG), endomysial, and deamidated gliadin peptide antibodies have become more significant in the celiac disease workup [2]. To date, the only available treatment for celiac disease is a strict gluten-free diet (GFD) for life [2]. Autoimmune thyroid diseases are polygenic and multifactorial disorders caused by immune system dysfunction [3]. Graves' disease (GD) and Hashimoto's thyroiditis (HT), both of which are characterized by the infiltration of autoreactive B and T lymphocytes into the thyroid parenchyma, constitute the AITD [3]. The incidence of Hashimoto's thyroiditis is roughly 3.5 cases per 1000 persons per year in women and 0.8 cases per 1,000 in males, with a prevalence of 2% to 3% of the population. Graves' disease affects 1% to 2% of women and 0.1% to 0.2% of males [3]. Epidemiological evidence points to an interplay between genetic susceptibility and environmental triggers (e.g., infection, diet, iodine, smoking, and alcohol) as the major mechanism for tolerance breakdown and disease development [3]. Cytokines and chemokines have also been demonstrated to play a role in the pathophysiology of HT and GD [3]. The principal autoantigens in Hashimoto's disease are thyroid peroxidase (TPO) and thyroglobulin (Tg), although these antibodies (anti-thyroid peroxidase (anti-TPO) antibody and anti-thyroglobulin (anti-Tg) antibody) are also found in 70% of Graves' disease patients [4]. Likewise, while thyroid-stimulating hormone receptor (TSHR) is the most common autoantigen in Graves' disease, it is also found in a small number of Hashimoto's disease patients [4]. GD usually manifests as thyrotoxicosis, whereas the clinical hallmark of HT is hypothyroidism. There is no compelling evidence linking CD to AITD due to extrinsic gluten-related factors such as age at first introduction, concurrent breastfeeding, duration of gluten exposure, gluten-free diet, etc. [5]. According to the existing research, the common genetic background is the key factor influencing the association's high incidence [5]. The diagnosis of CD may happen before or after the diagnosis of AITD [5]. The prevalence of AITD among CD patients, in particular, is well documented. However, the incidence of CD in AITD patients has received less attention and requires further investigation. The aim of this review article is to study the genetic overlap between AITD and CD, the prevalence of celiac disease in AITD, management, and prevention of these two conditions coexisting.

## Review

Genetic overlap

**Figure 1:**
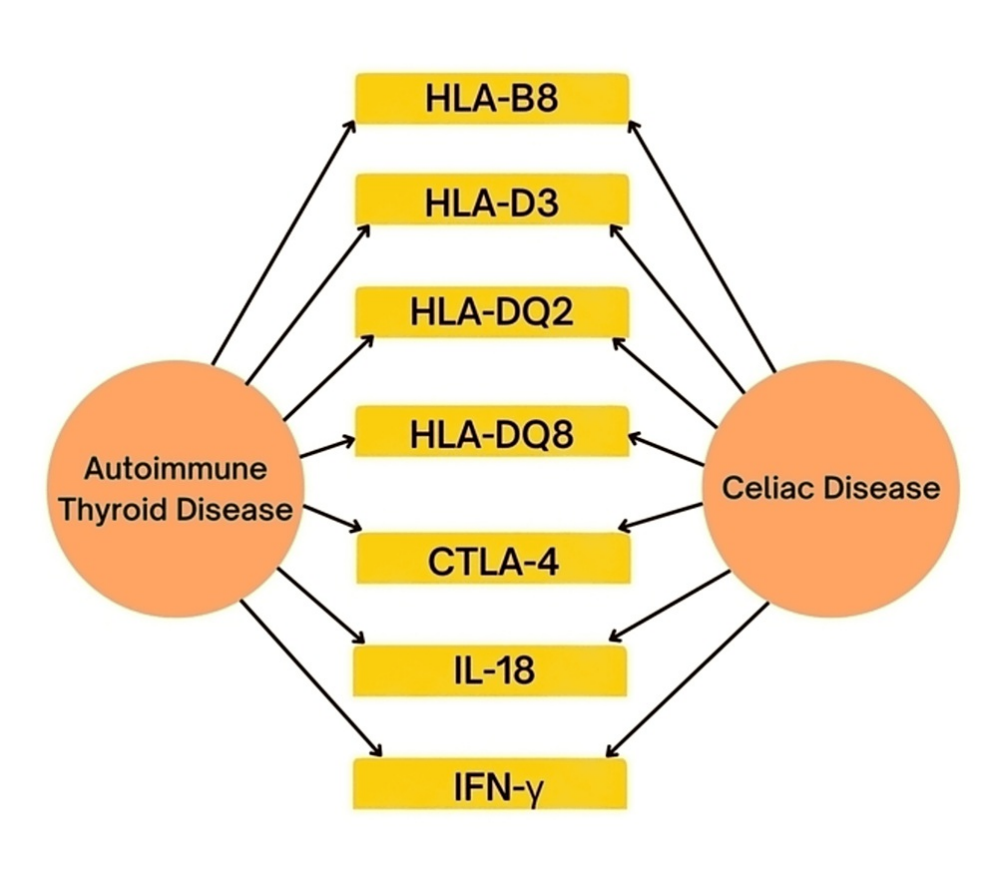
Shared genes between celiac disease and autoimmune thyroid disease. HLA: human leukocyte antigen, CTLA: cytotoxic T-lymphocyte-associated antigen, IL: interleukin, IFN: interferon.

The coexistence of AITD and CD is assumed to be partially attributable to a shared genetic susceptibility (Figure 1).

Many of these genes are components of the major histocompatibility complex (MHC). In 1986, Lorini et al. compared human leukocyte antigen (HLA) typing in a girl with a normal female karyotype affected by celiac disease and Graves' disease to that of a 15-year-old female described by Ruch et al. with Down's syndrome who acquired celiac disease, Graves' disease, and type 1 diabetes mellitus. Both HLA-B8 and HLA-DR3 antigens were detected in both patients, showing that these antigens are connected with an increased prevalence of autoimmune endocrine diseases and celiac disease, implying that both individuals have a genetic predisposition to these diseases [6]. Another study in 1999 included 62 adult patients who had more than one autoimmune endocrinologic disease. The HLA-DQ2 or DQ8 haplotypes appeared to explain some of the links between celiac disease and autoimmune diseases, as these celiac-type haplotypes were found in 69% of the study subjects [7]. In addition, several of them exhibited minor mucosal alterations that were consistent with the early stages of celiac disease [7]. Badenhoop et al. recruited 105 individuals with autoimmune thyroid diseases (81 with GD and 24 with HT) and 95 of their first-degree relatives. The authors assessed the prevalence of IgA autoantibodies to tTG (tTG-Ab) in the subjects and correlated the observations with HLA DQ status. There was an 8% prevalence of tTG-Ab among the HT patients investigated, whereas no tTG-Ab was discovered among the GD patients. Six of the relatives of the patients with GD, on the other hand, exhibited slightly or clearly elevated tTG-Ab, and three of them had the HLA DQ2 specificity. Five of the tTG-Ab positive relatives had HLA DQ2 and/or DQ8. A patient with both HLA DQ2 and HLA DQ8 had triple autoimmunity (type I diabetes mellitus, GD, and CD) [8]. On analyzing the HLA DQ of 271 people with Graves' disease (GD) and 65 people with Hashimoto's thyroiditis, it was found that endocrine autoimmunity has a shared immunogenetic basis; susceptibility is imparted by DQA1*0501 as well as an arginine at position 52 of DQA1 alleles [9]. Green et al. used an empirical logistic technique to analyze the correlation of HLA antigens with celiac disease and thyrotoxicosis. The relative risk of A1 with celiac disease was 1.5, whereas B8 was 6.8, implying that B8 was significantly more important to the disease's prevalence than A1. The relative risks for the correlations between thyrotoxicosis and A1 and B8, respectively, were 1.5 and 2.2. Only the association with B8 was significant for celiac disease and thyrotoxicosis [10]. Also, sharing comparable HLA genotypes may help explain the vital link between IgA deficiency and CD [3]. Although there is a weak association between HLA-DQ2, DQ8, and Hashimoto's thyroiditis, the relationship between HLA-DQ2 and Graves' disease is less evident [3]. CD and AITD have also been equated outside the HLA region to the gene encoding cytotoxic T-lymphocyte-associated antigen-4 (CTLA-4), a potential gene for thyroid autoimmunity susceptibility. Hunt et al. discovered a substantial link between the CTLA4 haplotype and celiac disease (P = 0.00067) [11]. A comprehensive study mapping the gene cluster found that variations in the CTLA4 30 area impact autoimmune responses in Graves' disease and Hashimoto’s thyroiditis [12]. In contrast to Grave’s disease, CD exhibits distinct correlations at the CTLA4 exon 1 single nucleotide polymorphism (SNP) +49G>A [13]. Valentino et al. observed that 10 of 14 Hashimoto's thyroiditis patients had genotypes that were consistent with CD (three patients had DQ heterodimer A1*0501, B1*0201, four patients had DRB1*04, and one patient had A1*0101, B1*0501, respectively). Six of the 14 patients had an increased density of γδ+ T-cell receptor-bearing intra-epithelial lymphocytes and evidence of mucosal T-cell activation, both of which are characteristic of CD. HLA genotypes linked with CD were also documented in four of these six individuals (three with DRB1*04, DQB1*03, and one with DQA1*0501, DQB1*02) [14]. Also, the common immunological T helper cell type 1 (Th1) pattern may concur with the pathophysiological basis of the association between CD and HT by polymorphisms in gene expression of both IL-18 and IFN-γ gene [15]. Single nucleotide polymorphisms (SNPs) in the IL-18 and IFN-genes might be exploited as biomarkers for the early detection of autoimmune illnesses such as CD and HT [15].

Clinical prevalence of celiac disease in autoimmune thyroid disease

Numerous screening investigations have found an increased prevalence of CD among people with AITD, but estimates vary greatly. A meta-analysis of 27 studies found that biopsy-confirmed CD was prevalent in 1.6% of AITD patients, and it was higher when compared to data from general population screening studies. Furthermore, the CD was shown to be more prevalent in children with AITD (6.2%) than in adults (2.7%) [16]. Collin et al. screened 83 Finnish patients with autoimmune thyroid diseases for celiac disease, using IgA-class reticulin and endomysium antibodies (EmA), IgA- and IgG-class gliadin antibody tests, and several biochemical tests for malabsorption. There were three asymptomatic celiac patients and one previously diagnosed celiac patient among the 83 individuals, accounting for a total incidence of 4.8% [17]. The findings revealed that the prevalence of subclinical celiac disease is higher in individuals with autoimmune thyroid diseases [17]. 

Sategna-Guidetti et al. discovered anti-endomysium antibodies (anti-EmA) in five of 152 autoimmune thyroid disease patients (3.3%), and celiac disease was verified histologically in all [18]. In an Italian study of 92 individuals with autoimmune thyroid disorders (47 with chronic autoimmune thyroiditis, 22 with Hashimoto's thyroiditis, and 23 with Graves' disease), four (4.3%) exhibited anti-gliadin and anti-endomysial positivity [19]. A similar study detected serum endomysial antibody positivity in five of 150 newly diagnosed AITD patients (3.3%) [20]. In a screening program, Berti et al. reported that six of 172 patients with autoimmune thyroiditis tested positive for anti-endomysium (3.4%), and five of these patients underwent intestinal biopsy, which revealed total villous atrophy [21]. In another study, IgA anti-tTG was identified in seven individuals with autoimmune thyroiditis, but IgA endomysial antibody was discovered in only six of them. Duodenal biopsy confirmed the diagnosis of celiac disease, demonstrating significant and mild villous atrophy in six and one of them, respectively. Interestingly, no IgA antibodies were detected in any of the euthyroid controls [22]. Larizza et al. investigated 90 children and adolescents with autoimmune thyroid disease. Celiac disease and DQA1*0501, DQB1*02 were detected in 7 (7.8%) of the patients, and the prevalence was one in every 13 individuals [23]. Meloni et al. investigated IgA-class and IgG-class anti-gliadin antibodies in 297 Sardinian individuals with autoimmune thyroid disease. Those who tested positive for either antibody had their EmA levels checked. Serum ferritin, folate, and vitamin B12 levels were determined, and patients were advised to have a jejunal biopsy if any two of the indicators were positive. Thirteen of the fourteen patients with at least two positive indicators underwent jejunal biopsies, and all demonstrated CD histological characteristics of celiac disease. The prevalence of CD in AITD patients was four times higher than in the general population (4.37% versus 1.06%, p<0.0001). There were no gastrointestinal complaints among the patients, although half of them showed hematinic deficits; six had iron deficiency, two had vitamin B12 deficiency, and none had folate deficiency. Furthermore, molecular typing of HLA class II alleles revealed an increase in the frequency of the extended haplotype DRB1*0301/DQA1*0501/DQB1*0201 [24]. In a cohort study of 100 patients with autoimmune thyroid disease, Mainardi et al. concluded that the prevalence of celiac disease was 2%. Remarkably, six months after beginning a gluten-free diet, these two celiac patients' serologic markers were undetectable [25]. In an Italian retrospective study, celiac disease was detected in 9.5% of 91 pediatric AITD patients. In addition, three (6.1%) of these children had first- and/or second-degree relatives with CD [26]. In a Turkish study, 101 children with AITD and 103 healthy children were evaluated for CD using the IgA anti-tTG antibody and total serum IgA. Eight children with AITD tested positive for the serological markers (7.9%), but none of the serum samples from healthy children were positive for IgA anti-tTG antibody. Also, in five patients (4.9%), subtotal villous atrophy was seen on performing intestinal biopsy [27]. In a similar study enrolling female teenagers, two (3%) of 66 patients with AITD were found to have CD [28]. A cross-sectional study in Jordan recruited 914 AITD patients, 117 of whom were seropositive for celiac disease (12.8%). About 39 (44.8%) of the 87 seropositive individuals who underwent duodenal biopsy exhibited positive histological findings of celiac disease [29]. Hadithi et al. assessed 104 Dutch patients with Hashimoto's thyroiditis, sixteen (15%) of whom tested positive for celiac serology. Five of them had documented villous atrophy and were diagnosed with celiac disease (4.8%; 95%CI 0.7-8.9). HLA-DQ2 and/or -DQ8 were found in all five and 53 Hashimoto's thyroiditis patients (50%; 95%CI 43-62) [30]. In an Iranian study published in 2012, eleven (2.4%) of 454 hypothyroid patients tested positive for celiac serology, and two patients with documented villous atrophy were diagnosed with classic CD (0.4%; 95%). HT was found in two patients with classic CD (0.6%; 95%) [31]. In an Indian screening study, Marwaha et al. discovered that levels of anti-tTG antibody increased with increasing titers of anti-TPO antibody [32]. Teixeira et al. reported a 1.2% prevalence of confirmed CD among Brazilian AITD patients, with a higher predisposition to the female gender [33].

Ch’ng looked specifically at the prevalence of CD in Graves' hyperthyroidism patients. Over a nine-month period, 111 patients with Graves' hyperthyroidism who visited the thyroid clinic were provided CD screening using an anti-gliadin antibody (AGA) and anti-tTG. Two of the patients had already been diagnosed with CD and were asymptomatic on a gluten-free diet. Graves' hyperthyroidism was diagnosed three and twenty-six years before celiac disease in these two patients. Three more CD patients were discovered during the screening, bringing the total prevalence to 4.5%, compared to 0.9% (1 of 115) of sex- and age-matched blood donor controls. There were no gastrointestinal symptoms in any of the five patients who had CD [34]. Similarly, Mankai et al. screened sera from 161 Tunisian Graves' disease patients and discovered that anti-EmA was positive in six of 161 (3.7%). All six of them were also positive for anti-tTG. The prevalence of biopsy-confirmed CD in this population was 1.86% (3/161) [35].

Effect of gluten-free diet on AITD

The strongest association between gluten consumption and thyroid destruction appears to be based on a molecular mimicry mechanism between gut and thyroid tissue transglutaminase [36]. There have been numerous studies looking into the effect of a gluten-free diet (GFD) on thyroid disease in patients with concomitant CD, but little is known about the effect of a GFD in AITDs in the absence of CD or celiac-related antibodies. An Italian multicenter study analyzing the thyroid function of 128 newly diagnosed CD patients before and one year after the introduction of a GFD observed that in some patients, a GFD could reverse thyroid abnormalities [37]. Valentino et al. found that three AITD patients with concomitant CD who followed a GFD for six months improved in symptoms related to hypothyroidism and thyroxine dosage. However, only one patient's anti-Tg and anti-TPO antibody levels changed after an 18-month follow-up [20]. A randomized controlled trial compared the effects of six months on a GFD (n = 16) to no dietary intervention (n = 18) in drug-naive women with HT. The GFD reduced levels of thyroid peroxidase and thyroglobulin antibodies increased 25-hydroxyvitamin D and improved the structure parameter inference approach-gain of thyroid (SPINA-GT) index, which correlated with changes in antibody titers. There was no effect on thyrotropin or free triiodothyronine levels [38]. In another recent study, participants were divided into two groups: those who took selenium with GFD and those who only took selenium supplementation without any other dietary intervention. At the end of the study, 37/50 (74%) of GFD participants achieved a euthyroid state, compared to 28/48 (58.3%) of the second group. Serum anti-TPO decreased more in the GFD group (by 49%) than in the control group (34%). Thyroid-stimulating hormone (TSH) and anti-TG levels decreased in both groups; however, gluten exclusion had an enhancing effect on anti-TPO levels [39]. In addition, Abbott et al. used a modified paleolithic diet on 12 HT patients in a dietary intervention study, while cereals, dairy products, and food additives were eliminated. After ten weeks of therapy, symptoms of the disease, as measured by the Medical Symptoms Questionnaire, decreased from 92 to 29 points, and quality of life, as measured by the 36-Item Short-Form Health Survey, improved significantly. However, thyroid function and anti-thyroid antibody serum concentrations did not improve, but the number of immune cells and high sensitivity C reactive protein decreased from 1.63 to 1.15 mg/L (−29%) [40]. Valentino et al. reported that clinical conditions improved among five AITD patients with CD, in accordance with the histological healing of the jejunal mucosa and the introduction of GFD, which resulted in a progressive reduction in the amount of thyroxine replacement therapy required and the titer of thyroid autoantibodies [20]. However, it is conceivable that a longer study time is required to reveal an effect of a GFD on AITD, as Ventura et al. reported that TPO antibodies were identified in only 76.9%, 46.1%, and 15.3% of CD patients with AITD at 6-, 12-, and 24-month GFD follow-ups, respectively [41].

On the other hand, a recent study indicated that a gluten-free diet did not seem to prevent the progression of the autoimmune process during a follow-up of one year on the levels of TPO antibodies detected in 10 newly diagnosed CD patients [42]. Furthermore, the thyroid volume decreased significantly compared to the patient group without CD (5.6 cm^3^ versus the initial 6.4 cm^3^), implying that thyroiditis continued to progress even after implementing a GFD [42]. Mainardi et al. observed an excellent clinical remission of CD six months after enrolling an AITD patient with CD on a gluten-free diet, with complete correction of the villous abnormalities and disappearance of all three serum celiac markers; however, an unexpected and progressive increase in the anti-thyroid antibodies titer was witnessed [25]. In a systematic study, Virili et al. discovered a few interesting findings. The need for T4 was found to be higher in patients with both HT and CD, and this effect could be avoided with a GFD. To achieve the target TSH value in patients with HT and CD who were not adhering to GFD, the therapeutic dose of T4 had to be increased by nearly 50%. The authors speculate that this could be explained by decreased absorption of levothyroxine in instances of untreated CD, as an increased need for levothyroxine was prevented by incorporating a GFD. However, reduced absorption capacity cannot account for the fact that patients with concomitant HT and CD had significantly higher TSH and significantly lower free T4 (p<0.0001) compared to patients with isolated HT (p<0.0001) before starting levothyroxine treatment [43]. Correspondingly, Zubarik et al., in a cohort study, demonstrated that patients who required higher doses (≥125 mcg/day) of levothyroxine to maintain a euthyroid state were more likely to have CD (p<0.001) [44]. Furthermore, Jiskra et al. reported that patients with chronic thyroiditis who received a dose of 125-200 mcg/day of levothyroxine had higher serum levels of IgA anti-gliadin antibodies than patients who received a lower dosage (50-100 mcg/day) [45]. Considering the studies mentioned above, it may be reasonable to assume that identifying and treating CD improves medication absorption for conditions such as HT. On the contrary, Sharma et al. reported in a screening study that there was no statistically significant difference in the thyroxine doses required for normalization of thyroid function in celiac autoimmunity positive and negative patients [46]. Similarly, Larizza et al. revealed no significant differences in autoantibody titer or amount of therapy based on the commencement of the GFD. However, the only patient who ceased L-thyroxine therapy was affected by CD [23]. Oderda et al. noticed decreased levels of celiac antibodies in children with CD while on a GFD for one to five years, but anti-TPO titers decreased in only two of six children and increased in the rest. According to the authors, gluten elimination may not be beneficial if thyroid autoimmunity has already developed [47]. Gluten exclusion for one year had some beneficial effects on autoimmune thyroiditis in 28.6% of the subjects in an interventional study by Rasheed et al. The authors did point out, however, that GFD compliance had no effect on the presence of antibodies as new anti-thyroid antibody-positive cases emerged [48]. 

Need for screening

Celiac disease meets the World Health Organization's (WHO) criteria for general population screening, but the proposal remains contentious [49]. Screening high-risk subjects, particularly patients with disorders commonly associated with CD, maybe the most feasible strategy due to the possible increased morbidity of one disease superimposed on the other [3]. According to the current European Society for Pediatric Gastroenterology, Hepatology, and Nutrition (ESPGHAN) guidelines, screening for CD in children with AITD is recommended [50]. As per the 2016 meta-analysis, all patients with AITD should be screened for CD due to the increased prevalence of the coexistence of these two disorders. The study suggests that patients with HT have celiac serological tests performed and that if any of the celiac serological tests are positive, the patients be investigated further with gastroduodenoscopy and duodenal biopsy [16]. In an Indian case-control study, Soni et al. identified that first-degree relatives of celiac disease patients have a threefold increased risk of developing AITD and associated thyroid dysfunction. The authors propose that first-degree relatives of CD patients be screened for both CD and AITD because most patients are asymptomatic [51]. However, we could not find any studies regarding screening relatives of AITD patients for CD. Ch'ng et al., revealing a 4.5% prevalence of CD in an outpatient population receiving GD treatment, advocated for routine screening [34]. Interestingly, Fanciulli et al. discovered no cases of celiac disease after screening 231 AITD patients in a clinical practice setting. Despite screening procedures performed in research-setting studies showing the prevalence of celiac disease in AITD patients to be approximately 4-15 times higher than in the general population, the researchers discourage the applicability of screening in clinical practice. As screening is a resource-intensive activity for both patients and clinicians, a critical assessment of the output of screening is recommended before its use in clinical practice [52]. An American study that excluded AITD patients with coexisting type 1 diabetes and Down syndrome discovered that the prevalence of CD in AITD was only 1.3%, similar to the general population. The authors concluded that the increased prevalence of CD in AITD was largely caused by enrichment with comorbidities and that screening for celiac disease may not be justified in the absence of these comorbidities [53]. Considering routine screening does not appear to be cost-effective, the findings of Virili et al. imply that an increased requirement for levothyroxine should prompt a search for an occult gastrointestinal disorder, which could help reveal an occult or atypical CD [43].

Table 1 gives details of the clinical studies showing the prevalence of celiac disease among patients affected by autoimmune thyroid disease.

**Table 1:** Summary of screening studies showing the prevalence of celiac disease among autoimmune thyroid disease patients. CD: celiac disease, AITD: autoimmune thyroid disease, HT: Hashimoto’s thyroiditis, GD: Grave’s disease, anti-TPO: anti-thyroid peroxidase.

References	Country	Population screened	Number of patients with CD	Prevalence of CD (%)
Collins et al. [17]	Finland	83 AITD patients	4	4.8
Sategna-Guidetti et al. [18]	Italy	152 AITD patients	5	3.3
Cuoco et al. [19]	Italy	47 Chronic immune thyroiditis patients, 22 HT patients, and 23 GD patients	4	4.3
Valentino et al. [20]	Italy	150 AITD patients	5	3.3
Berti et al. [21]	Italy	172 AITD patients	5	3.5
Volta et al. [22]	Italy	220 AITD patients	7	3.2
Larizza et al. [23]	Italy	90 AITD pediatric patients	6	7.8
Meloni et al. [24]	Italy	297 AITD patients	13	4.4
Mainardi et al. [25]	Italy	100 AITD patients	2	2.0
De Martino et al. [26]	Italy	91 AITD pediatric patients	9	9.9
Sari et al. [27]	Turkey	101 AITD pediatric patients	5	5.0
Sahin et al. [28]	Turkey	66 AITD pediatric patients	2	3.0
Farahid et al. [29]	Jordan	914 HT patients	39	4.3
Hadithi et al. [30]	Netherlands	104 HT patients	5	4.8
Mehrdad et al. [31]	Iran	454 Hypothyroid patients	2	0.4
Ch’ng et al. [34]	United Kingdom	111 GD patients	5	4.5
Mankai et al. [35]	Tunisia	161 GD patients	3	1.9
Marwaha et al. [32]	India	1154 Subjects (577 anti-TPO positive patients and 577 age- and sex-matched controls)		6.9 Among cases, 3.5 among controls
Teixeira et al. [33]	Brazil	254 AITD patients (143 GD patients and 111 HT patients)	3	1.2
Zubarik et al. [44]	United States of America	498 Hypothyroid patients	9	1.8
Sattar et al. [53]	United States of America	302 AITD patients	7	2.3

Limitations

Celiac disease is a multisystem disease that has several associations, including Sjogren syndrome, systemic sclerosis, rheumatoid arthritis, and idiopathic inflammatory myopathies. However, it is beyond the scope of this paper to consider all of those aspects. AITD may also be influenced by nutritional factors such as iodine, zinc, and selenium levels. However, we did not take them into account.

## Conclusions

The principal aim of this review article was to consolidate the most relevant literature associating celiac disease with autoimmune thyroid diseases. There appears to be a significant overlap in genetic variables between CD and AITD. However, more rigorous clinical studies would be required to complement genetic discoveries. In a longitudinal strategy, AITD patients need to be systematically phenotyped and screened for CD autoantibodies, which would offer precise information regarding the prevalence of co-occurrence and the chronological order of occurrences in individual patients. The rising prevalence of CD in AITD patients is extensively documented. Also, the majority of the studies on the effects of a gluten-free diet in AITD patients in the presence of concomitant CD have been shown to cause beneficial effects in the management of both diseases. However, a few studies have demonstrated no significant changes in the autoantibody titer in spite of following a gluten-free diet. Although individuals with AITD must be considered high-risk for CD, the need for routine screening is debatable due to cost-effectiveness and a lack of a significant number of cases reported in prior screening trials. Furthermore, given the frequency of subclinical presentations of CD and AITD, it is challenging to identify the concurrent occurrence of both these diseases precociously. Due to the multisystem nature of celiac disease, a multidisciplinary approach is recommended and should be integrated into the diagnostic algorithm of autoimmune thyroid diseases to ensure that patients with concurrent AITD and CD receive the best possible management. In order to adapt focused clinical therapy for the management of these two concurrent disorders, we urge more future investigations targeting AITD patients for genes related to celiac disease and the subclinical and clinical prevalence of celiac disease.
